# Polygenic risk of type 2 diabetes is associated with incident vascular dementia: a prospective cohort study

**DOI:** 10.1093/braincomms/fcad054

**Published:** 2023-03-06

**Authors:** Elin Dybjer, Atul Kumar, Katarina Nägga, Gunnar Engström, Niklas Mattsson-Carlgren, Peter M Nilsson, Olle Melander, Oskar Hansson

**Affiliations:** Department of Clinical Sciences Malmö, Lund University, Jan Waldenströms gata 35, SE-21428 Malmö, Sweden; MultiPark: Multidisciplinary Research focused on Parkinson's disease, Lund University, Box 117, SE-22100 Lund, Sweden; Clinical Memory Research Unit, Department of Clinical Sciences Malmö, Lund University, Skånes universitetssjukhus, VE Minnessjukdomar, SE-20502 Malmö, Sweden; Department of Clinical Sciences Malmö, Lund University, Jan Waldenströms gata 35, SE-21428 Malmö, Sweden; Clinical Memory Research Unit, Department of Clinical Sciences Malmö, Lund University, Skånes universitetssjukhus, VE Minnessjukdomar, SE-20502 Malmö, Sweden; Department of Acute Internal Medicine and Geriatrics, Linköping University, SE-58183 Linköping, Sweden; Department of Clinical Sciences Malmö, Lund University, Jan Waldenströms gata 35, SE-21428 Malmö, Sweden; MultiPark: Multidisciplinary Research focused on Parkinson's disease, Lund University, Box 117, SE-22100 Lund, Sweden; Clinical Memory Research Unit, Department of Clinical Sciences Malmö, Lund University, Skånes universitetssjukhus, VE Minnessjukdomar, SE-20502 Malmö, Sweden; Brain Injury After Cardiac Arrest Research Group, Lund University, Box 117, SE-22100 Lund, Sweden; WCMM – Wallenberg Centre for Molecular Medicine, Lund University, Sölvegatan 19, BMC D11, SE-22184 Lund, Sweden; Department of Clinical Sciences Malmö, Lund University, Jan Waldenströms gata 35, SE-21428 Malmö, Sweden; EpiHealth: Epidemiology for Health Strategic Research Area, Lund University, SUS Malmö, Jan Waldenströms gata 35, SE-20502 Malmö, Sweden; Department of Clinical Sciences Malmö, Lund University, Jan Waldenströms gata 35, SE-21428 Malmö, Sweden; EpiHealth: Epidemiology for Health Strategic Research Area, Lund University, SUS Malmö, Jan Waldenströms gata 35, SE-20502 Malmö, Sweden; Department of Emergency and Internal Medicine, Skåne University Hospital, SE-20502 Malmö, Sweden; EXODIAB: Excellence in Diabetes Research in Sweden, Lund University, Box 117, SE-22100 Lund, Sweden; MultiPark: Multidisciplinary Research focused on Parkinson's disease, Lund University, Box 117, SE-22100 Lund, Sweden; Clinical Memory Research Unit, Department of Clinical Sciences Malmö, Lund University, Skånes universitetssjukhus, VE Minnessjukdomar, SE-20502 Malmö, Sweden

**Keywords:** Alzheimer’s disease, Mendelian randomization, polygenic risk score, type 2 diabetes, vascular dementia

## Abstract

Type 2 diabetes and dementia are associated, but it is unclear whether the two diseases have common genetic risk markers that could partly explain their association. It is also unclear whether the association between the two diseases is of a causal nature. Furthermore, few studies on diabetes and dementia have validated dementia end-points with high diagnostic precision. We tested associations between polygenic risk scores for type 2 diabetes, fasting glucose, fasting insulin and haemoglobin A_1c_ as exposure variables and dementia as outcome variables in 29 139 adults (mean age 55) followed for 20–23 years. Dementia diagnoses were validated by physicians through data from medical records, neuroimaging and biomarkers in cerebrospinal fluid. The dementia end-points included all-cause dementia, mixed dementia, Alzheimer’s disease and vascular dementia. We also tested causal associations between type 2 diabetes and dementia through two-sample Mendelian randomization analyses. Seven different polygenic risk scores including single-nucleotide polymorphisms with different significance thresholds for type 2 diabetes were tested. A polygenic risk score including 4891 single-nucleotide polymorphisms with a *P*-value of <5e-04 showed the strongest association with different outcomes, including all-cause dementia (hazard ratio 1.11; Bonferroni corrected *P* = 3.6e-03), mixed dementia (hazard ratio 1.18; Bonferroni corrected *P* = 3.3e-04) and vascular dementia cases (hazard ratio 1.28; Bonferroni corrected *P* = 9.6e-05). The associations were stronger for non-carriers of the Alzheimer’s disease risk gene *APOE* ε4. There was, however, no significant association between polygenic risk scores for type 2 diabetes and Alzheimer’s disease. Furthermore, two-sample Mendelian randomization analyses could not confirm a causal link between genetic risk markers of type 2 diabetes and dementia outcomes. In conclusion, polygenic risk of type 2 diabetes is associated with an increased risk of dementia, in particular vascular dementia. The findings imply that certain people with type 2 diabetes may, due to their genetic background, be more prone to develop diabetes-associated dementia. This knowledge could in the future lead to targeted preventive strategies in clinical practice.

## Teaser

Dybjer *et al.* report that polygenic risk of type 2 diabetes is associated with dementia risk, in particular vascular dementia. Associations were strongest for non-carriers of the gene *APOE* ε4. However, two-sample Mendelian randomization analyses did not support a causal relationship between type 2 diabetes and vascular dementia.

## Graphical Abstract

**Graphical Abstract:**
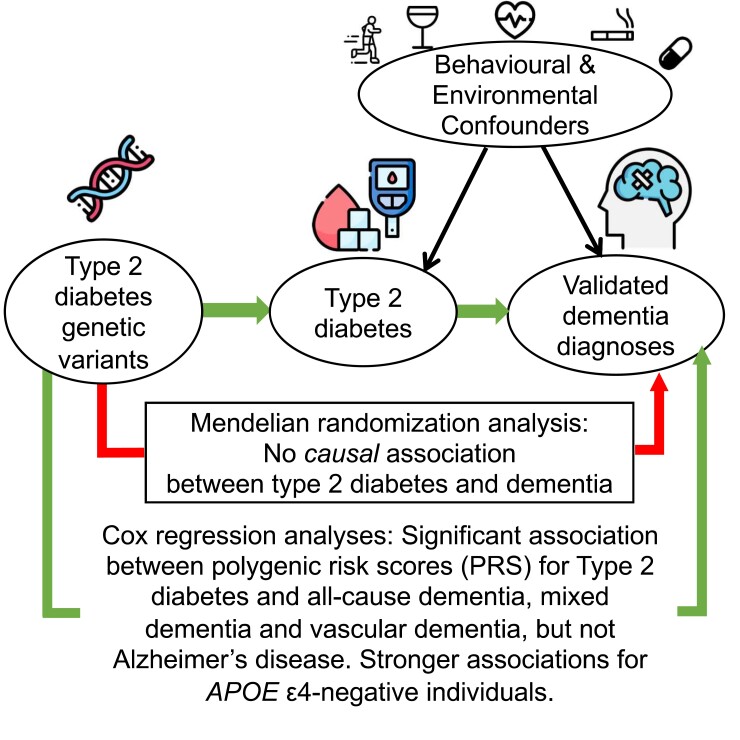

## Introduction

Type 2 diabetes is associated with an increased risk of dementia in epidemiological studies with a relative risk (RR) of 2.5 for vascular dementia and 1.5 for Alzheimer’s disease.^1^ Most interventional studies based on anti-diabetes drugs have, however, so far not been successful in preventing cognitive decline in people with type 2 diabetes.^2,3^ One reason may be that certain groups of people with type 2 diabetes may benefit more from preventive treatment. This could potentially concern individuals that are more susceptible to developing dementia within the group of people with type 2 diabetes. It is therefore of value to identify genetic risk markers of type 2 diabetes that could also be predictive of dementia.

A way of studying a wide set of genetic risk markers that are associated with a trait in relation to an outcome is through construction of polygenic risk scores (PRS)^4^ that summarize an individual’s propensity to a trait. Most PRS studies have so far not been able to identify an association between type 2 diabetes, or associated phenotypes such as insulin resistance, and dementia risk.^5,6^ Two exceptions are a recent study where a PRS for type 2 diabetes was linked to vascular dementia^7^ and a study on patients with major depressive disorder (but without type 2 diabetes) where there was a link between PRS for type 2 diabetes and impaired cognitive functioning.^8^ A limitation of some previous studies on genetic risk markers of type 2 diabetes and dementia is that they have not used validated dementia end-points, but instead other outcomes related to dementia or cognitive decline, such as genetic risk markers of dementia,^5^ cognitive test results^6^ or dementia based on registered ICD diagnosis codes.^7,9^

Observational studies have shown that vascular pathophysiological factors are likely to be the most predominant in causing cognitive decline,^10^ but that metabolic pathways also may play a role. It has for instance been proposed that certain traits that are closely associated with the phenotype of type 2 diabetes, such as levels of glucose, haemoglobin A_1c_ (HbA_1c_) and insulin, could be partly responsible for the association between type 2 diabetes and dementia.^11^ Whether genetic susceptibility to these traits could predict dementia is therefore also an important phenomenon to investigate further.

Furthermore, it is unclear whether there is a causal link between type 2 diabetes and dementia. Some studies using Mendelian randomization (MR) have investigated this matter but have not yet found any causality within the association between the two diseases.^12–15^ On the other hand, other MR studies have found evidence of causal associations between closely related phenotypes to diabetes and dementia, for example between fasting plasma glucose and risk of Alzheimer’s disease^16^ or unspecified dementia,^9^ between HbA_1c_ levels and impaired visual memory^13^ and between insulin resistance and Alzheimer’s disease.^16^

The ‘aim’ of this study was to investigate associations between genetic risk markers of type 2 diabetes, fasting glucose, fasting insulin and HbA_1c_ as exposure variables and dementia diagnoses as outcome variables. The dementia diagnoses were validated by physicians and included the subtypes mixed dementia (Alzheimer’s disease in combination with significant cerebrovascular co-pathology), Alzheimer’s disease (without significant cerebrovascular co-pathology) and vascular dementia. We also tested the influence of the Alzheimer’s disease risk gene variant apolipoprotein E ε4 (*APOE* ε4) burden on the associations, as this may further increase dementia risk in persons with type 2 diabetes.^17^ A secondary ‘aim’ was to investigate causal associations between type 2 diabetes and dementia through MR analyses.

## Material and methods

### Participants

We used data from the Malmö Diet and Cancer Study (MDCS) baseline examination carried out during 1991–94, a prospective population-based cohort study of 30 446 participants from Malmö, Southern Sweden. The mean age at baseline was 55 years, and the participation rate was 41%. Reasons for non-participation and loss to follow-up have previously been described.^18^

A self-administered questionnaire was distributed with questions on demographics, previous diseases, lifestyle habits and medications. A health examination was carried out in which blood pressure (mmHg), weight (kg) and height (m) were measured and fasting blood samples were drawn. Educational level was classified into elementary school (<8 years of education), upper secondary school (9–12 years), higher education/university (≥12 years) or missing data. Smoking was classified as ‘never-smoker’, ‘past’, ‘current’ smoking or missing data. Physical activity level was calculated into a score based on data on number of minutes per week of activity, type of activity and an activity-specific factor and was then divided into tertiles with an added category for missing data. Alcohol was classified into ‘no consumption’, ‘consumption below risk level’ (<9 standard units of 12 g/week for women or 14 units for men), ‘consumption above risk level’ or missing data. A history of cardiovascular disease was defined as reporting having had a coronary event or a stroke.

Genotyping was carried out when technically possible in 29 451 of the blood samples using the Illumina GSA v1 genotyping array. Due to missing data on age, blood sample number or a mismatch in ID and blood sample number, 72 participants were excluded, and a further 240 due to failing the genetic data quality control (QC) as described below. The final study population thus arrived at 29 139 participants.

### Genetic QC

Standard QC steps were performed for single-nucleotide polymorphism (SNP)-level filter. High-quality variants [autosomal, non-monomorphic, bi-allelic variants with Hardy–Weinberg equilibrium (HWE) *P* > 5 × 10^−8^ and with a call rate of >99%] were used. Multi-allelic variants and SNPs with a data imputation score < 0.2 have been excluded as part of post-imputation QC, and genotype calls with a posterior likelihood < 0.9 have been set to fail (i.e. hard-called). SNPs with a genotyping rate of >0.9 were retained. SNPs with minor allele frequency (MAF) ≥ 5% were taken for the analysis. Supplementary sensitivity analyses using MAF ≥ 1% were also performed regarding PRS for type 2 diabetes and dementia outcomes, to check the possible impact of rare genetic variants on the results. Multi-dimensional scaling was done using PLINK 2^19^ to create principal components in genetic analyses to account for ancestry.

### PRC calculation

Using an *r*2 < 0.1 threshold over 1000 kb sliding windows (index threshold and clumped SNPs of *P* < 1), linkage-disequilibrium (LD) clumping was performed using PLINK’s clump function. LD clumping ensures that PRS calculation is not overloaded by large blocks of correlated SNP sets. The PRS was determined for each subject by summing up the effective number of alleles (0, 1, 2) of the SNPs weighted by the natural logarithm of their respective odds ratio (OR). The default formula for PRS calculation in PLINK is:


$$PRS_{j}=\;\frac{\sum_{i}^{N}\;S_{i}*G_{ij}}{P*\;M_{j}}$$

where the effect size of SNP *i* is *S_i_*; the number of effect alleles observed in sample *j* is *G_ij_*; the ploidy of the sample is *P* (is generally 2 for humans); the total number of SNPs included in the PRS is *N*; and the number of non-missing SNPs observed in sample *j* is *M_j_*. If the sample has a missing genotype for SNP *i*, then the population MAF multiplied by the ploidy (MAF_i_ ∗ *P*) is used instead of *G_ij_*.

Publicly accessible summary statistics from reported genome-wide association studies (GWAS) (not overlapping with our data set) of type II diabetes,^20^ HbA_1c_,^21^ fasting glucose^22^ and fasting insulin^23^ were used to define PRS. We iterated over a variety of *P*-values (0.05–5 × 10^−8^) to evaluate the appropriate *P*-value threshold: *P* = 0.05 (PRS 1), *P* = 5 × 10^−3^ (PRS 2), *P* = 5 × 10^−4^ (PRS 3), *P* = 5 × 10^−5^ (PRS 4), *P* = 5 × 10^−6^ (PRS 5), *P* = 5 × 10^−7^ (PRS 6) and the GWAS-level significance thresholds of *P* = 5 × 10^−8^ (PRS 7) creating models named PRS 1–7.

### Clinical type 2 diabetes

Type 2 diabetes at baseline was defined using combined data from the Diabetes 2000 Register, the National Diabetes Register (NDR), the HbA_1c_ registry, the Swedish Cause of Death Register, the Swedish National Patient Register (NPR), the Swedish Prescribed Drug Register and the Alla Nya Diabetiker I Skåne (ANDIS) Register, as well as cohort data from the Malmö Preventive Project and the Malmö Diet and Cancer Study including data from blood samples, self-reported diabetes and drug use. Type 2 diabetes was then classified as (i) having a diabetes diagnosis in any of the sources; (ii) not having specified type 1 diabetes, gestational diabetes, secondary diabetes, MODY diabetes or other specified diabetes types; and (iii) not having self-reported insulin treatment as only diabetes treatment at baseline. After exclusion criteria were applied, 1119 participants were defined as having prevalent type 2 diabetes at baseline.

### Validated dementia end-points

The validation process of dementia diagnoses in the MDCS cohort has previously been described.^24,25^ The validity was assessed using data from medical records by trained medical doctors at the Memory Clinic, Skåne University Hospital in Malmö. National Patient Register (NPR) diagnoses of dementia from the baseline examination until 31 December 2014 were obtained (*n* = 2206), covering 99% of inpatient medical diagnoses during the study period, and from 2001 also outpatient diagnoses,^26^ although not diagnoses from primary care facilities. The participants were thus followed for 20–23 years after the baseline examination in 1991–94.

Out of 2206 dementia cases, 1697 (77%) were diagnosed by a specialist in cognitive disorders, 1904 (86%) had data on symptoms, 1894 (86%) on cognitive test results, and 2080 (94%) on CT, MRI or both. CSF analyses were available for 839 (38%) of the participants. The NPR diagnoses were through this information re-classified in accordance with the Diagnostic and Statistical Manual of Mental Disorders, Fifth Edition (DSM-V).^27^

The final number of dementia cases after validation was 2039 out of which 578 were mixed dementia (Alzheimer’s disease + vascular dementia), 510 were vascular dementia, 598 were Alzheimer’s disease, and the remaining 353 were other forms of dementia (not analysed in this study).

### Statistical analyses

Statistical analyses were performed using IBM SPSS version 25 for Mac OSX, R version 4.1.1, R studio version 1.4.1717 and Python version 3.9.6. Independent-samples *t*-tests and chi-square tests were used to test for differences in cohort characteristics between participants with and without type 2 diabetes.

### Clinical type 2 diabetes and dementia risk

First, epidemiological associations between clinical type 2 diabetes and incident dementia were examined through Cox regression analyses. We used two adjustment models based on the fact that demographic factors and cardiovascular factors can affect the risk of both type 2 diabetes and dementia^28^ and also that cardiovascular factors can be seen as potential mediators of the association.^29^ ‘Model 1’ was therefore adjusted for age, sex and education and ‘Model 2’ for age, sex, education, smoking, alcohol consumption, physical activity, systolic blood pressure (SBP), body mass index (BMI), apolipoprotein B/apolipoprotein A-ratio (ApoB/ApoA-ratio), history of cardiovascular disease (stroke or coronary event), use of anti-hypertensive medication and use of lipid-lowering treatment.

As *APOE* ε4 genotype is a strong genetic risk marker of Alzheimer’s disease^30^ and is also associated with atherosclerosis and cardiovascular disease^31,32^ that are prevalent in people with type 2 diabetes, we tested for interactions with *APOE* ε4 in the analyses (‘Model 1’ with interaction term of *APOE* ε4 genotype ∗ type 2 diabetes). An equivalent analysis stratified for *APOE* ε4 genotype was performed when the interaction term was significant.

We also considered risk of Alzheimer’s disease as a competing event in *APOE* ε4 carriers in the analysis with type 2 diabetes as predictor and all-cause dementia as outcome. For the *APOE* ε4-positive group, we therefore carried out a Fine–Gray subdistribution hazard model^33^ with type 2 diabetes as exposure, all-cause dementia (except Alzheimer’s disease cases) as outcome and Alzheimer’s disease as competing event, adjusted for age, sex and education.

### Association between PRS for type 2 diabetes and clinical type 2 diabetes

To investigate whether genetic risk of type 2 diabetes and clinical type 2 diabetes were associated, we also carried out binary logistic regression analyses with PRS 1–7 (as defined above) for type 2 diabetes as exposure and clinical type 2 diabetes at baseline as outcome.

### Association between PRS for type 2 diabetes, fasting glucose, insulin and HbA_1c_ as exposure, and dementia as outcome

We then performed Cox regression analyses to investigate associations between PRS 1–7 for (i) type 2 diabetes, (ii) fasting glucose, (iii) fasting insulin and (iv) HbA_1c_ as exposure and time to first dementia event as outcome. The analyses were adjusted for age, sex and education in ‘Model 1’ and the same factors and additionally ApoE-ε2 and ApoE-ε4 genotype (0, 1 or 2 alleles) in ‘Model 2’. We also tested interactions between *APOE* ε4 status (0 or 1–2 alleles) and PRS 1–7 on the association with dementia. For analyses where interaction terms were significant, we then stratified for *APOE* ε4 status in additional Cox regression analyses. We also carried out a competing risk analysis in *APOE* ε4-positive participants with Alzheimer’s disease as competing event, PRS 1–7 as exposure and all-cause dementia (except for Alzheimer’s disease) as outcome (Fine–Gray subdistribution hazard model as above). A sensitivity analysis using MAF ≥ 1% was also performed for the results of PRS for type 2 diabetes and dementia outcomes.

### MR analyses

Finally, we carried out two-sample MR analyses using summary statistics on genetic risk markers of type 2 diabetes from the same reference data set.^20^ We used the MR methods MR Egger, simple mode, weighted mode, weighted median and inverse variance-weighted MR to test whether type 2 diabetes shared genetic effects with any of the dementia subtypes. The MR test was based on 243 independent variants (used as instrumental variables) of type 2 diabetes with a genome-wide significant association at *P* < 0.5 ∗ 10^−8^. We could not perform MR analyses for the exposure variables fasting glucose, insulin and HbA_1c_, as there were not enough SNPs with genome-wide significance.

### Ethics approval and consent to participate

The study was approved by the Ethical Committee of Lund University, Sweden (MDC baseline examination LU-51–90, MDC Re-examination Dnr.532/2006 and for genetic analyses in 2009 (Dnr 2009/682). Written informed consent to participate was obtained from all participants.

## Results

Characteristics of the MDCS cohort are presented in Table 1. The group with type 2 diabetes had a higher mean age (on average 3.6 older than the group without type 2 diabetes), a higher proportion of men, a lower average educational level and a higher prevalence of cardiovascular risk factors than the group without type 2 diabetes. For example, average lipid levels, systolic blood pressure and BMI were higher, as well as proportions of previous smokers and of previous cardiovascular disease. Proportions of current smokers and high alcohol intake were however higher in people without diabetes. There was no significant difference between the groups as regards *APOE* status. Out of people with type 2 diabetes, 11% were diagnosed with dementia during the follow-up period compared to 6.8% of the control group, for which all dementia subtypes were less prevalent.

**Table 1:** Characteristics of the Malmö Diet and Cancer cohort and comparisons between participants with type 2 diabetes and controls

	Total study sample (*n* = 29 139)	No type 2 diabetes (*n* = 28 020)	Type 2 diabetes (*n* = 1119)	*P* for difference between groups
**Baseline data**				
Age, mean (SD)	58.1 (7.61)	57.9 (7.61)	61.3 (6.69)	**<0**.**001**
Gender (% women/men)	60.4/39.6	60.9/39.1	46.7/53.3	**<0**.**001**
Education (%)				
Missing data	6.2	6.1	8.8	
<8 years	42.0	39.1	48.1	**<0**.**001**
9–12 years	35.0	33.1	27.5	
≥13 years	22.9	21.8	15.6	
Smoking (%)				
Missing data	6.0	5.9	8.5	
Never	26.5	26.7	21.6	**<0**.**001**
Former	31.9	31.7	37.8	
Current	35.6	35.7	32.1	
Alcohol consumption (%)				
Missing data	8.0	7.9	11.2	
No consumption	15.6	15.2	25.2	**<0**.**001**
Below risk level	42.5	42.6	40.4	
Above risk level	33.9	34.3	23.2	
Physical activity level (%)				
Missing data	6.9	6.7	9.7	
Low	31.0	30.9	35.7	**<0**.**001**
Medium	31.0	31.3	25.6	
High	31.0	31.1	29.0	
Systolic blood pressure, mean (SD)**^a^**	141.2 (20.0)	140.8 (20.0)	150.3 (19.7)	**<0**.**001**
BMI, mean (SD)**^a^**	25.8 (4.04)	25.7 (3.97)	28.6 (4.68)	**<0**.**001**
S-ApoB/ApoA-ratio, mean (SD)	0.71 (0.22)	0.70 (0.22)	0.82 (0.27)	**<0**.**001**
History of **cardiovascular disease** (%)	3.0	2.8	8.1	**<0**.**001**
Use of anti-hypertensive treatment (%)	17.4	16.4	41.0	**<0**.**001**
Use of lipid lowering treatment (%)	3.0	2.8	10.2	**<0**.**001**
***APOE* ε4 (%)**				
**Non-carrier**	69.9	69.8	69.9	
**Heterozygous**	27.4	27.5	27.4	0.422
**Homozygous**	2.7	2.7	2.7	
***APOE* ε2** (%)				
**Non-carrier**	85.0	85.0	84.6	
**Heterozygous**	14.4	14.4	14.6	0.634
**Homozygous**	0.6	0.6	0.8	
**Outcomes**				
All-cause dementia, ***n*** (%)	2039 (7.0)	1914 (6.8)	125 (11.2)	**<0**.**001**
Mixed dementia, ***n*** (%)	578 (2.0)	541 (1.9)	37 (3.3)	**0**.**001**
Vascular dementia, ***n*** (%)	510 (1.8)	464 (1.7)	46 (4.1)	**<0**.**001**
Alzheimer’s disease, ***n*** (%)	598 (2.1)	571 (2.0)	27 (2.4)	0.386

Significant p-values (*p* < 0.05) are highlighted in bold text.

a Valid data for *n* = 29 091.

Epidemiological associations between clinical type 2 diabetes and time to first dementia event are presented in Table 2, calculated through Cox proportional hazard modelling. Compared with participants without diabetes, participants with prevalent type 2 diabetes at baseline had a 1.46 times higher hazard ratio (HR) of all-cause dementia, a 1.61 times higher HR of mixed dementia and a 1.84 times higher HR of vascular dementia after full adjustment. Type 2 diabetes was, however, not associated with a higher HR of Alzheimer’s disease. There was a significant negative interaction between *APOE* ε4 status and type 2 diabetes on the risk of dementia for all-cause dementia (HR for interaction term 0.62, *P* = 0.011), as well as Alzheimer’s disease (HR 0.46, *P* = 0.047), but not for mixed dementia or vascular dementia. When stratifying for *APOE* ε4 status, associations between type 2 diabetes and all forms of dementia were stronger for non-carriers of *APOE* ε4. In *APOE* ε4 carriers, there was only a significant association between type 2 diabetes and vascular dementia in ‘Model 1’, whereas there were no other significant associations between type 2 diabetes and dementia end-points. However, type 2 diabetes was significantly associated with all-cause dementia (except for Alzheimer’s disease) in *APOE* ε4 carriers when taking into account the competing risk of Alzheimer’s disease, with a subhazard ratio (sHR) of 1.49, 95% confidence interval (CI) 1.05–2.11, *P* = 0.025.

**Table 2:** Multivariable Cox proportional hazards modelling of years from birth to first dementia event with clinical type 2 diabetes as predictor

	Model 1		Model 2	
	HR (95% CI)	*P*	HR (95% CI)	*P*
**Total cohort (*n* = 29 139)**				
All-cause dementia	1.61 (1.34–1.92)	**<0**.**001**	1.46 (1.20–1.77)	**<0**.**001**
Mixed dementia	1.83 (1.32–2.54)	**<0**.**001**	1.61 (1.12–2.30)	**0**.**010**
Vascular dementia	2.25 (1.67–3.03)	**<0**.**001**	1.84 (1.32–2.58)	**<0**.**001**
Alzheimer’s disease	1.16 (0.79–1.70)	0.464	1.26 (0.84–1.89)	0.272
**No *APOE* ε4 (*n* = 20 359)**				
All-cause dementia	1.99 (1.58–2.52)	**<0**.**001**	1.83 (1.42–2.36)	**<0**.**001**
Mixed dementia	2.23 (1.42–3.48)	**<0**.**001**	2.00 (1.23–3.27)	**0**.**006**
Vascular dementia	2.44 (1.67–3.56)	**<0**.**001**	2.16 (1.43–3.26)	**<0**.**001**
Alzheimer’s disease	1.85 (1.07–3.20)	**0**.**027**	1.82 (1.00–3.31)	**0**.**049**
***APOE* ε4+ (*n* = 8780)**				
All-cause dementia	1.26 (0.94–1.68)	0.117	1.16 (0.84–1.60)	0.369
Mixed dementia	1.45 (0.87–2.40)	0.152	1.22 (0.69–2.16)	0.488
Vascular dementia	1.96 (1.17–3.27)	**0**.**010**	1.59 (0.87–2.92)	0.136
Alzheimer’s disease	0.90 (0.52–1.56)	0.704	1.09 (0.62–1.91)	0.774

Analyses are presented for the total cohort and then stratified for *APOE* ε4 genotype (0 or 1–2 alleles). ‘Model 1’ is adjusted for age, sex and education. ‘Model 2’ is adjusted for age, sex, education, smoking, alcohol consumption, physical activity level, SBP, BMI, blood pressure medication, lipid lowering treatment, ApoB/ApoA-ratio and history of cardiovascular disease. Significant *P*-values are highlighted in **bold** text.

In Supplementary Table 1, associations between PRS 1–7 for type 2 diabetes and clinical type 2 diabetes are presented. All associations were significant. The area under the curve (AUC) in receiver operating curves (ROC) was around 0.65 for all PRS.

**Figure 1:**
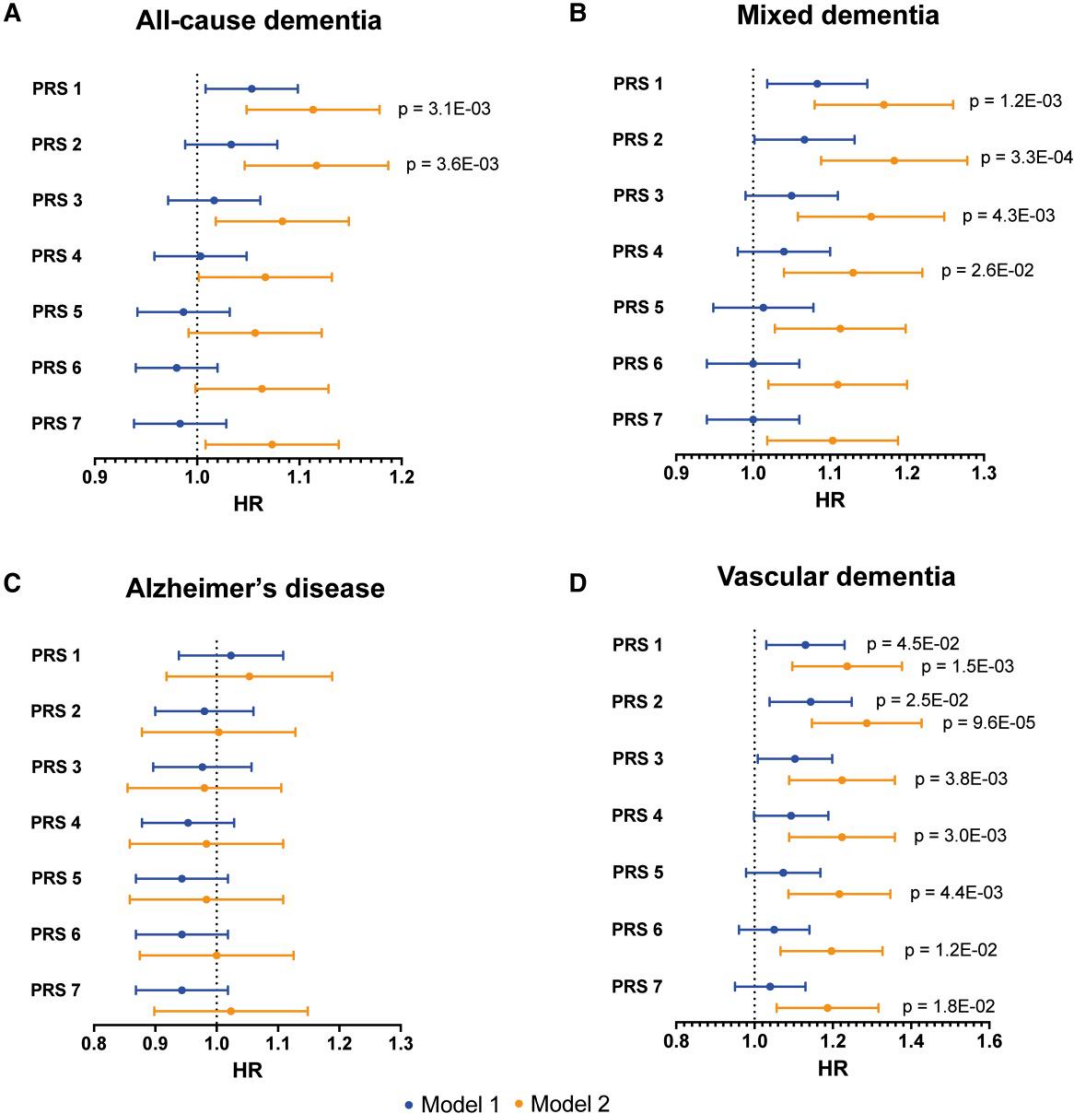
**Forest plots of polygenic risk scores (PRS) for type 2 diabetes as exposure and incident dementia over time as outcome, calculated through Cox proportional hazards modelling.** The horizontal lines represent hazard ratios (HR) with 95% confidence intervals (CI) of time to incident dementia for the different PRS 1–7 (*P*-value thresholds 5e-2 through 5e-8). Results are shown for the dementia types: (**A**) all-cause dementia, (**B**) mixed dementia, (**C**) Alzheimer’s disease and (**D**) vascular dementia. ‘Model 1’ is adjusted for age, gender and education. ‘Model 2’ is adjusted for age, gender, education, *APOE* ε2 and *APOE* ε4 status (0 or 1–2 alleles). Only *P*-values significant after Bonferroni correction are shown.

**Figure 2:**
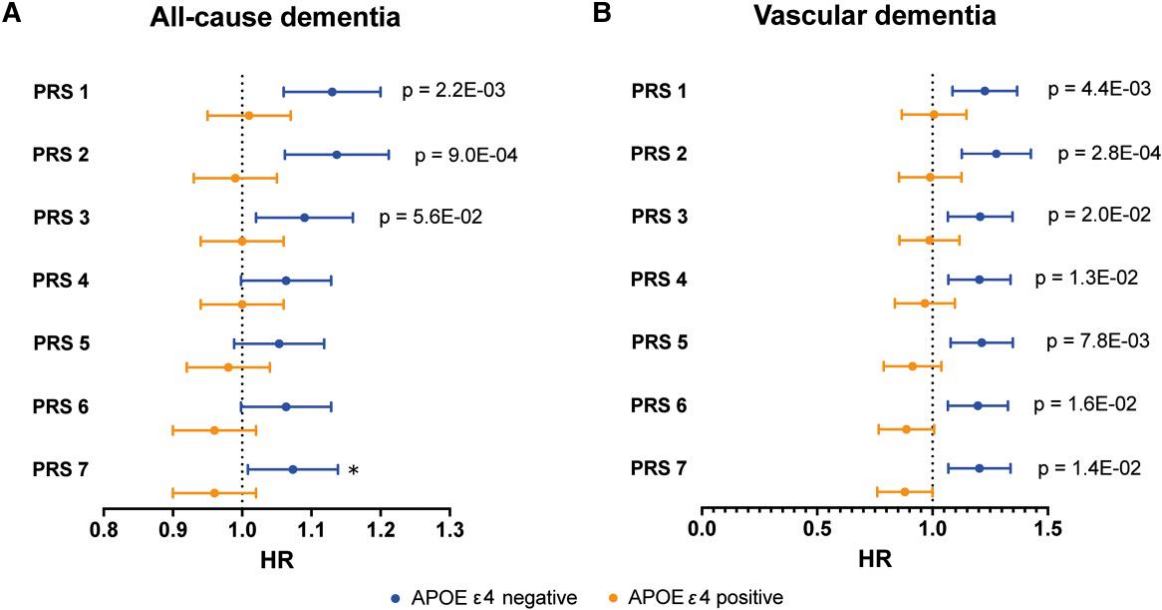
**Stratified for *APOE* status (ε2 and ε4 1–2 alleles): forest plots of polygenic risk scores (PRS) for type 2 diabetes as exposure and incident dementia over time as outcome, calculated through Cox proportional hazards modelling.** The horizontal lines represent hazard ratios (HR) with 95% confidence intervals (CI) of time to incident dementia for the different PRS 1–7 (*P*-value thresholds 5e-2 through 5e-8). Results are shown for the dementia types: (**A**) all-cause dementia and (**B**) vascular dementia. ‘Model 1’ is adjusted for age, gender and education. ‘Model 2’ is adjusted for age, gender, education, *APOE* ε2 and *APOE* ε4 status (0 or 1–2 alleles). Only *P*-values significant after Bonferroni correction are shown.

In Fig. 1 and Supplementary Tables 2–5, results of Cox regression analyses with PRS 1–7 for type 2 diabetes (standardized) as exposure and time to first dementia event (all-cause dementia, mixed dementia, Alzheimer’s disease and vascular dementia, respectively) as outcome are presented, adjusted for age, sex and education in ‘Model 1’ and for age, sex, education and *APOE* burden (APOE ε4 and ε2 count) in ‘Model 2’. There were no significant associations between any of the PRS for type 2 diabetes and the dementia end-points using adjustment ‘Model 1’. In ‘Model 2’, however, PRS 1 (variants with *P*-value < 5e-02) and PRS 2 (variants with *P*-value < 5e-03) were significantly associated with all-cause dementia (HR of 1.11 for both PRS, Bonferroni corrected *P*-value = 3.9e-03 and 3.6e-03 respectively, Supplementary Table 2). PRS 1–4 were also significantly associated with mixed dementia with the strongest association for PRS 2 (HR of 1.18, Bonferroni corrected *P* = 3.3e-04, Supplementary Table 3). No significant associations were found between PRS for type 2 diabetes and Alzheimer’s disease (Supplementary Table 4). All the type 2 diabetes PRS were however significantly associated with risk of vascular dementia, out of which PRS 2 showed the strongest association with a HR of 1.28 (Bonferroni corrected *P*-value = 9.6e-05) (Supplementary Table 5). There was a significant interaction between *APOE* ε4 and the association between PRS 1 and 2 for type 2 diabetes and all-cause dementia (Supplementary Table 2) as well as between PRS 1–7 for type 2 diabetes and vascular dementia (Supplementary Table 5). Stratified analyses for *APOE* ε4 were therefore carried out between PRS 1–7 for type 2 diabetes and all-cause and vascular dementia. These are shown in Fig. 2 and Supplementary Table 6. For non-carriers of *APOE* ε4, higher PRS 1–3 significantly increased the hazard of all-cause dementia and all PRS increased the hazard of vascular dementia (average HR per SD of PRS 1.2, *P* < 0.002). For carriers of *APOE* ε4, none of these associations were significant. In subdistribution hazard models with PRS 1–7 as exposure, all-cause dementia (except Alzheimer’s disease) as outcome and Alzheimer’s disease as competing event, the associations were also non-significant (see last two columns of Supplementary Table 2).

**Figure 3:**
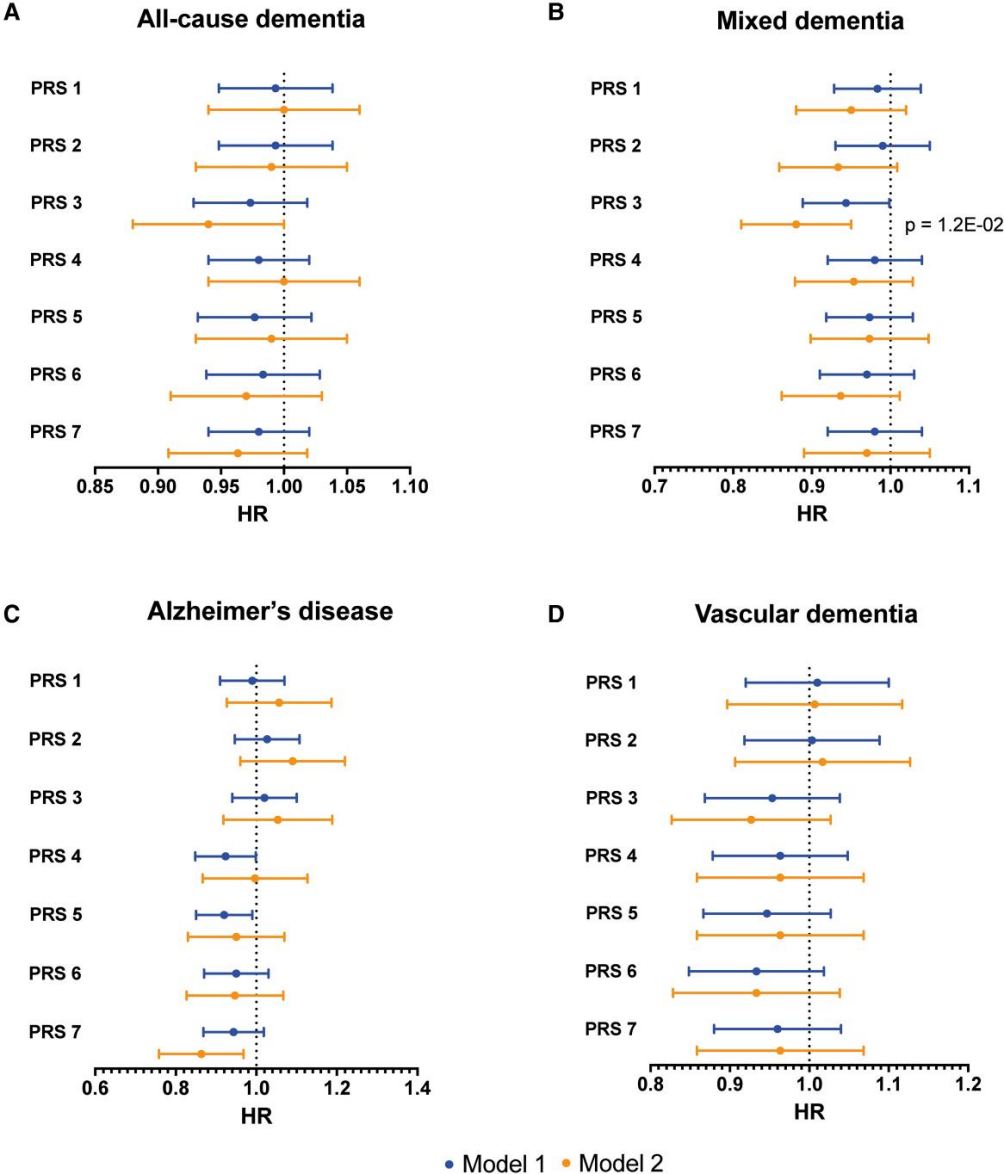
**Forest plots of polygenic risk scores (PRS) for fasting insulin as exposure and incident dementia over time as outcome, calculated through Cox proportional hazards modelling.** The horizontal lines represent hazard ratios (HR) with 95% confidence intervals (CI) of time to incident dementia for the different PRS 1–7 (*P*-value thresholds 5e-2 through 5e-8). Results are shown for the dementia types: (**A**) all-cause dementia, (**B**) mixed dementia, (**C**) Alzheimer’s disease and (**D**) vascular dementia. ‘Model 1’ is adjusted for age, gender and education. ‘Model 2’ is adjusted for age, gender, education, *APOE* ε2 and *APOE* ε4 status (0 or 1–2 alleles). Only *P*-values significant after Bonferroni correction are shown.

Results of Cox proportional hazard modelling with PRS 1–7 for HbA_1c_, fasting glucose and fasting insulin as exposure variables and dementia as outcome are presented in Supplementary Tables 7–10 (HbA_1c_), 11–14 (fasting glucose) and 15–18 (fasting insulin). The results of PRS 1–7 for fasting insulin are also visualized in Fig. 3. There were no significant associations between PRS for HbA_1c_ or fasting glucose and dementia outcomes. However, there was a significant negative association between PRS 3 for fasting insulin and mixed dementia (HR of 0.88, Bonferroni corrected *P*-value = 1.2e-02). Fasting insulin PRS 3 was also significantly negatively associated with all-cause dementia (HR = 0.94; uncorrected *P*-value = 4.3e-02) and PRS 7 with Alzheimer’s disease (HR = 0.86; uncorrected *P*-value = 1.6e-02), although not after Bonferroni correction.

In Supplementary Results 1, Supplementary Figs 1 and 2 and Supplementary Table 19, sensitivity analyses with MAF ≥ 1% for the analyses of PRS for type 2 diabetes and dementia outcomes are shown. Using this level did not largely change the results compared with the main results using MAF ≥ 5%.

Finally, in Supplementary Table 20, two-sample MR analyses of genetic variants of type 2 diabetes (shared effects of all 243 genetic variants with genome-wide significance) as exposure and dementia end-points as outcome are presented. No significant causal associations between type 2 diabetes and dementia were found. We also tested the casual association between type 2 diabetes and dementia within the *APOE* ε4-negative subgroup using two-sample MR. However, we did not find any significant causality effect between the two traits using different two-sample MR methods (Supplementary Table 21).

## Discussion

In this population-based study, PRS of type 2 diabetes were associated with all-cause dementia, mixed dementia and vascular dementia, but not with Alzheimer’s disease. Likewise, clinical type 2 diabetes was associated with the same outcomes during the follow-up period of 20–23 years. The associations between PRS for type 2 diabetes and all-cause dementia as well as vascular dementia were stronger in the part of the population that did not have the *APOE* ε4 genotype. We also noted that PRS 2 [a PRS generated using a weighted score of less significant variants (*P* < 0.005)] was most significant among all the PRS in most of the models. This suggests that the combined effect of these less significant variants might be responsible for causing dementia. In two-sample MR analyses, however, no causal associations were found between type 2 diabetes and dementia outcomes. Furthermore, there were negative associations between fasting insulin and the outcomes all-cause dementia and Alzheimer’s disease, although the associations did not remain after Bonferroni correction. PRS of fasting glucose and HbA_1c_ were, however, not significantly associated with dementia outcomes.

### Findings in relation to other studies

Our study confirms what a number of epidemiological studies have shown previously, which is that type 2 diabetes is associated with an increased risk of dementia, in particular vascular dementia.^1^ Similar to a study published very recently (in November 2022),^7^ we also identify a link between PRS for type 2 diabetes and dementia (all-cause dementia and vascular dementia), which other previous studies have not been able to do.^5,6^ One reason for this could be that we have well validated data on dementia diagnoses, while other genetic studies previously have used other ways of defining dementia or cognitive impairment (e.g. genetic risk of Alzheimer’s disease,^5^ cognitive test results^6^ or register-based dementia diagnoses^9^). Register-based dementia diagnoses in particular can be lacking in diagnostic accuracy.^34^ Cognitive test results have advantages in the sense that they can report precise measurements of cognitive ability in different domains. However, validated dementia diagnoses as they are defined in our study have the advantage that they are formed through combined data from different sources to find a more reliable diagnosis. Furthermore, genetic risk of Alzheimer’s disease is a precise and quantitative measure but does not take into account the clinical phenotype of interest (i.e. dementia).

In our study, we did not find an association between type 2 diabetes and Alzheimer’s disease. Many studies have found a link between the two diseases, whereas others, for instance one on neuropathological post-mortem findings of Alzheimer’s disease, have not.^35^ It has been hypothesized that diabetes is primarily responsible for vascular changes that can be present in Alzheimer’s disease.^36^ Hence, since our Alzheimer’s disease end-point was defined as Alzheimer’s disease without visible cerebrovascular brain changes on MRI scans in most cases, this might be a reason for not finding a significant association with Alzheimer’s disease in this group.

### No causal link between diabetes and dementia?

In line with previous MR studies,^12–15^ we could not detect any causality in the association between type 2 diabetes and dementia. The fact that we could not detect a causal link should, however, be interpreted with caution because the power was limited in the sense that a negative finding cannot be interpreted as absence of effect.

It remains an enigma why a causal association between type 2 diabetes and dementia cannot be found despite there being so many hypotheses of underlying mechanisms behind the association (increased cerebrovascular burden due to diabetes-related factors,^37–42^ as well as effects on neurodegenerative processes by for instance glucose levels,^43^ insulin resistance^44^ and advanced glycation end products^11^). However, a possible explanation is that the heterogenicity in the pathogenesis of type 2 diabetes makes direct causal associations between the two diseases hard to detect. Furthermore, only 10% of the SNPs that explain the heritability of type 2 diabetes are currently known.^45^ Hence, similar studies in the future may have a better chance of detecting causal associations.

It is also possible that there are elements of type 2 diabetes that cause dementia (e.g. hyperglycaemia), but that cannot be detected through two-sample MR analyses with stringent *P*-value thresholds for the included genetic risk variants. Improved knowledge of underlying genetic risk variants of hyperglycaemia and other related traits may help future studies to investigate causal inference of these traits with dementia. Another explanation for the epidemiological association between type 2 diabetes and dementia could be that there are instead causal pathways between type 2 diabetes and phenotypes close to dementia. For example, one study employed MR to investigate effects of predisposition to type 2 diabetes, hyperglycaemia, insulin resistance and beta-cell dysfunction on risk of stroke subtypes and related cerebrovascular phenotypes. The study detected causal effects of type 2 diabetes and hyperglycaemia on large artery and small vessel stroke as well as brain atrophy.^46^ In other words, there are many pathways that need further investigation through studies including genetic risk markers of different phenotypes related to both type 2 diabetes and to dementia and cognitive impairment.

### Potential role of *APOE* ε4 carriership for the association?

Our findings interestingly show a strong association between polygenic risk of type 2 diabetes, as well as clinical type 2 diabetes, and vascular dementia, although not Alzheimer’s disease, in non-carriers of *APOE* ε4. These are very similar findings to a recent study with similar study design as ours.^7^ This other study had a larger study sample size than ours, but did not analyse the dementia diagnoses longitudinally using time to event as outcome, and did not have validated dementia diagnoses. The findings in our two studies are in contrast to a meta-analysis that reported that *APOE* ε4 carriers are more vulnerable to diabetes-associated dementia.^17^

Since we could not detect a significant association between type 2 diabetes and all-cause dementia in *APOE* ε4 carriers, we hypothesized that this might be because carriers of *APOE* ε4 have a competing risk of Alzheimer’s disease and are therefore exposed to a relatively lower risk of diabetes-associated dementia. In an analysis taking the competing risk of Alzheimer’s disease into account, type 2 diabetes was significantly associated with all-cause dementia in *APOE* ε4 carriers, which shows that our hypothesis might be true. In an equivalent analysis but using PRS 1–7 as exposure, the association between PRS 1–7 and all-cause dementia was, however, still non-significant in *APOE* ε4 carriers. This might be due to lack of statistical power or simply due to lack of causal effects between genetic risk markers of type 2 diabetes and dementia in this subgroup of people.

### PRS for fasting insulin associated with dementia risk

We found a protective effect of polygenic risk of higher fasting insulin on Alzheimer’s disease and all-cause dementia (although most of the estimates did not survive Bonferroni correction). We have not found any other study that examined this particular hypothesis. However, epidemiological studies have shown an excess risk of dementia in people with lower serum insulin levels^47^ as well as higher levels of insulin resistance.^16^ Insulin resistance in the brain can also lead to negative effects on whole body metabolism according to current research.^44^ Further studies would therefore be of value to investigate the role of underlying genetic factors for the association between insulin levels or insulin resistance and cognitive decline.

### Strengths and limitations

Strength of our study was that the dementia end-points were systematically validated by physicians at the Memory Clinic in Malmö. Register data on dementia diagnoses were hereby re-evaluated through assessment of medical records, neuro-imaging data and CSF biomarkers when available. However, a limitation was that the dementia subtypes after validation were still not classified as definite in many cases (a definite diagnosis in 59% of Alzheimer’s disease cases, 51% of mixed dementia cases and 32% of vascular dementia cases), using a stringent threshold for definite diagnosis (all key data from medical records and neuroimaging reports available). The proportion of diagnoses that were classified as ‘probable’, i.e. the second highest level on a four-level scale (a vast majority of the data indicating a specific diagnosis), or definite was higher; 88% for Alzheimer’s disease, 91% for mixed dementia and 76% for vascular dementia.^25^ Taken together with the fact that 76% of the dementia cases were diagnosed by a specialist in cognitive disorders (which is unusually high in this population), as well as the fact that 40% of the register-based diagnoses were changed after validation, we believe that these aspects indicate a relatively high diagnostic precision in general of the dementia diagnoses.

Other limitations of the study were the relatively low participation rate at baseline (41%) and the study sample size that is somewhat smaller than other studies investigating the same research questions. Follow-up for validated dementia diagnoses was restricted to the end of 2014 for logistical reasons. The study population, dominated by White Europeans, is derived from Skåne in Southern Sweden, why the findings may not be pertinent to broader populations at risk of type 2 diabetes and dementia, or other ethnicities. Furthermore, we could not perform external validation due to the non-availability of relevant data including validated dementia end-points.

### Clinical implications

Our study suggests that certain people with type 2 diabetes may, due to their genetic background, be more prone to develop diabetes-associated dementia than other people. If more studies can replicate these findings, it may be possible to identify patient groups that could benefit from preventive strategies to mitigate cognitive decline. It remains to be discovered which preventive strategies that in that case would be the most effective for these patient groups. Lifestyle modification (including physical activity, diet modification and cognitive training)^48^ and treatment with glucagon-like peptide-1 (GLP-1) analogues^49^ are alternative strategies that have been proposed to be able to help slow down the process of diabetes-associated dementia, but more studies are needed to confirm these hypotheses.

## Conclusions

In conclusion, the results of this population-based study show that there are genetic risk markers of type 2 diabetes that are also predictive of incident dementia, in particular vascular dementia. No causal associations between type 2 diabetes and dementia were, however, found in MR analyses. However, as type 2 diabetes is a heterogenous disease with a multifactorial aetiology, it is possible that there are still factors that cause dementia among the contributing pathophysiological factors of type 2 diabetes, which could be identified in further studies.

## Supplementary Material

fcad054_Supplementary_Data

## Data Availability

The data that support the findings of this study are available from the Steering Committee of the MDCS, but restrictions apply to the availability of these data, which were used under license for the current study, and so are not publicly available. Data are however available from the authors upon reasonable request and with permission of Mr. Anders Dahlin, data manager (e-mail:anders.dahlin@med.lu.se).

## Abbreviations

ApoB/ApoA-ratio =: apolipoprotein B/apolipoprotein A-ratio
*APOE* ε4 =: apolipoprotein E ε4
BMI =: body mass index
GLP-1 =: glucagon-like peptide-1
GWAS =: genome-wide association study
HbA_1c_ =: haemoglobin A_1c_
HWE =: Hardy–Weinburg equilibrium
LD =: linkage disequilibrium
MAF =: minor allele frequency
MR =: Mendelian randomization
PRS =: polygenic risk score
QC =: quality control
SNP =: single-nucleotide polymorphism
