# Supplementary material for: Polygenic risk of type 2 diabetes is associated with incident vascular dementia: a prospective cohort study
Source: Brain Commun. 2023 Mar 6;5(2):fcad054. doi: 10.1093/braincomms/fcad054 (PMC10118265; doi:10.1093/braincomms/fcad054)
Supplement: fcad054_Supplementary_Data [file fcad054_supplementary_data.pdf]

Supplementary material

Supplementary Results

Comparison of PRS models with variants at Minor allele frequency of 0.01 and 0.05

We also generated type 2 diabetes PRS models with variants at minor allele frequency (MAF) of 0.01, instead of 0.05 as presented in the main manuscript. The aim was to observe if the involvement of rare variants alters the dementia risk prediction compared to the PRS models having variants at MAF 0.05. PRS *Model 1* (adjusted for age, gender and education) and PRS *Model 2* (adjusted for the same factors as model 1 and additionally ApoE-ε2 and ApoE-ε4 genotype) with variants at MAF 0.01 did not show any significant difference when compared to the *Models 1* and *2* with MAF 0.05 (**Supplementary Figure 1 and 2**). However, there were more statistically significant results when we used MAF 5% than when we used 1%. This may be because rare variants in our samples did not have a high effect size in the type 2 diabetes GWAS summary statics[1]. As seen from the results (**Supplementary Table 19**), the number of SNPs significantly changed for PRS 1 when using this threshold, followed by PRS 2, but for the other PRSs, the change was nominal. As they were non-significant with less effect size, they did not contribute much to the PRS score.

Reference

1. Mahajan A, Taliun D, Thurner M, et al. Fine-mapping type 2 diabetes loci to single-variant resolution using high-density imputation and islet-specific epigenome maps. *Nature Genetics*. 2018/11/01 2018;50(11):1505-1513. doi:10.1038/s41588-018-0241-6

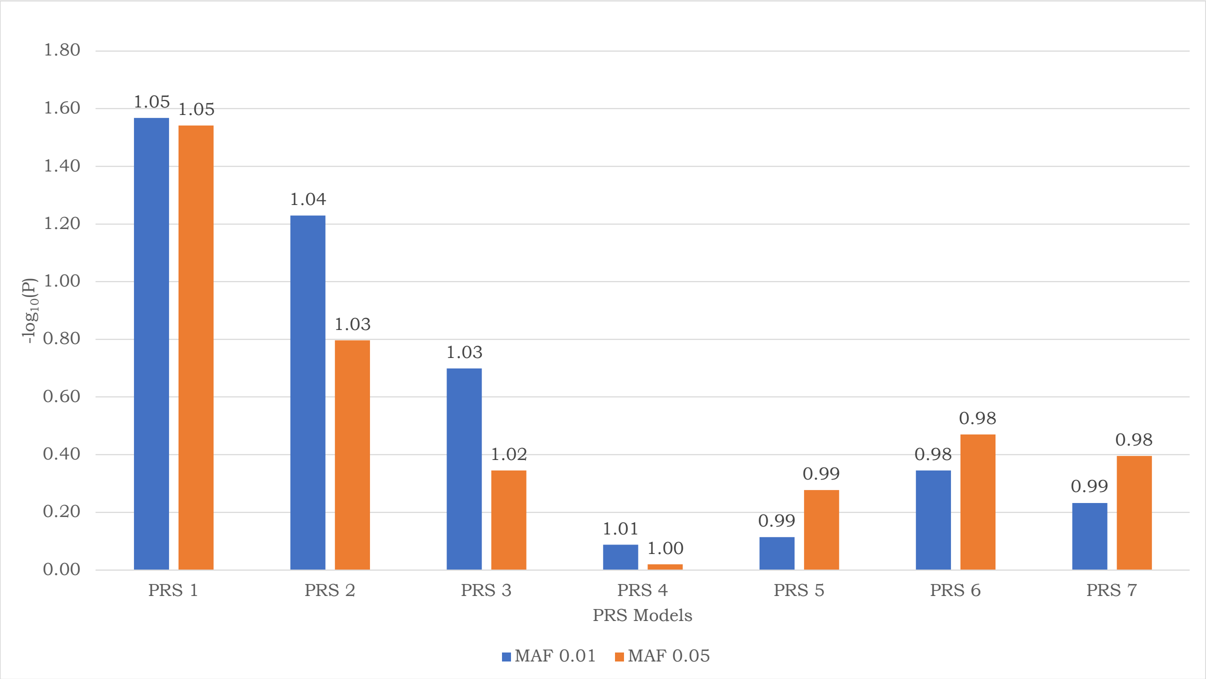

Supplementary Figure 1. Comparison of PRS models with Minor allele frequency (MAF) of 0.01 and 0.05 for *Model 1* for Multivariable Cox regression analyses with PRS 1-7 for type 2 diabetes as exposure and all-cause dementia as outcome. The bars represent the negative logarithm of the p-value for each association (blue representing MAF 0.01 and orange MAF 0.05). The hazard ratios (HR) are displayed above each bar for the corresponding association.

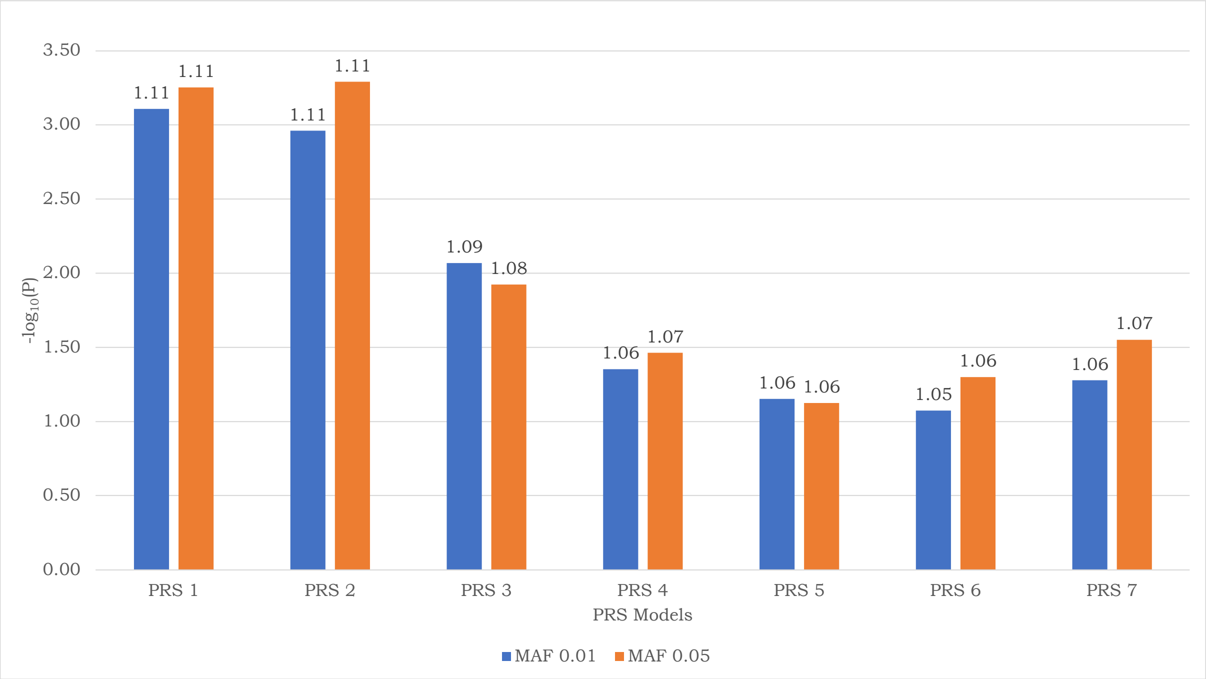

Supplementary Figure 2. Comparison of PRS models with Minor allele frequency (MAF) of 0.01 and 0.05 for *Model 2* for Multivariable Cox regression analyses with PRS 1-7 for type 2 diabetes as exposure and all-cause dementia as outcome. The bars represent the negative logarithm of the p-value for each association (blue representing MAF 0.01 and orange MAF 0.05). The hazard ratios (HR) are displayed above each bar for the corresponding association.

Supplementary Tables

Supplementary Table 1. Binary logistic regression analyses of associations between PRS 1-7 for type 2 diabetes (standardised) and clinical type 2 diabetes. Odds ratios (OR) of the associations, corresponding p-values, Bonferroni corrected p-values, as well as area under the curve (AUC) with 95% confidence intervals are shown (L95_AUC = lower bound, U95_AUC = upper bound).

PRS	P-value threshold for PRS-score	OR	P for OR	Bonferroni-p	AUC	L95_AUC	U95_AUC
**PRS 1**	5,00E-02	1,65	<0.001	1,40E-15	0,71	0.46	0.58
**PRS 2**	5,00E-03	1,78	<0.001	1,40E-15	0,72	0.54	0.66
**PRS 3**	5,00E-04	1,76	<0.001	1,40E-15	0,72	0.53	0.65
**PRS 4**	5,00E-05	1,71	<0.001	1,40E-15	0,71	0.49	0.61
**PRS 5**	5,00E-06	1,68	<0.001	1,40E-15	0,71	0.48	0.60
**PRS 6**	5,00E-07	1,66	<0.001	1,40E-15	0,71	0.46	0.58
**PRS 7**	5,00E-08	1,59	<0.001	1,40E-15	0,70	0.42	0.54

Supplementary Table 2. Multivariable Cox regression analyses of associations between PRS 1-7 for type 2 diabetes (standardised) and all-cause dementia. Hazard ratios (HR), p-values, number of SNPs (nSNP), 95% confidence intervals for hazard ratios (L95_HR and U95_HR for lower and upper bounds), and Bonferroni-corrected p-values are shown for each PRS-score. In Model 2 p-values for interaction between APOE ε4 (0 or 1-2 alleles) and the association between PRS 1-7 and all-cause dementia are also shown. A competing risk regression analysis was also performed, corresponding to the analyses in Model 1, for which subdistribution hazard ratios (sHR) with 95% confidence intervals and p-values are shown.

	HR	P	nSNP	L95_HR	U95_HR	Bonferroni-p	Interaction-p	sHR (95%CI)	sHR_p
**MODEL 1**								1 (0.93, 1.09)	9,10E-01
**PRS 1**	1,05	2,87E-02	16405	1,01	1,10	2,01E-01		0.97 (0.90, 1.05)	4,90E-01
**PRS 2**	1,03	1,60E-01	4891	0,99	1,08	1		0.98 (0.91, 1.06)	6,40E-01
**PRS 3**	1,02	4,51E-01	1795	0,97	1,06	1		0.99 (0.92, 1.08)	8,60E-01
**PRS 4**	1,00	9,54E-01	882	0,96	1,05	1		0.96 (0.89, 1.04)	3,30E-01
**PRS 5**	0,99	5,28E-01	536	0,94	1,03	1		0.93 (0.86, 1.00)	6,30E-02
**PRS 6**	0,98	3,38E-01	357	0,94	1,02	1		0.93 (0.86, 1.00)	6,10E-02
**PRS 7**	0,98	4,02E-01	253	0,94	1,03	1			
**MODEL 2**									
**PRS 1**	1,11	5,57E-04	16405	1,05	1,18	3,90E-03	4,36E-02		
**PRS 2**	1,11	5,12E-04	4891	1,05	1,19	3,58E-03	2,11E-02		
**PRS 3**	1,08	1,19E-02	1795	1,02	1,15	8,33E-02	1,07E-01		
**PRS 4**	1,07	3,43E-02	882	1,00	1,13	2,40E-01	6,43E-02		
**PRS 5**	1,06	7,48E-02	536	0,99	1,12	5,24E-01	5,80E-02		
**PRS 6**	1,06	5,01E-02	357	1,00	1,13	3,51E-01	1,57E-02		
**PRS 7**	1,07	2,80E-02	253	1,01	1,14	1,96E-01	1,10E-02		

Model 1**:** adjusted for age, sex and education.
Model 2**:** adjusted for age, sex, education, APOE ε2 count, and APOE ε4 count (0, 1 or 2 alleles).

Supplementary Table 3. Multivariable Cox regression analyses of associations between PRS 1-7 for type 2 diabetes (standardised) and mixed dementia. Hazard ratio (HR), p-value, number of SNPs (nSNP), 95% confidence intervals for hazard ratios (L95_HR and U95_HR for lower and upper bounds), and Bonferroni-corrected p-values are shown for each PRS-score. In Model 2 p-values for interaction between APOE ε4 (0 or 1-2 alleles) and the association between PRS 1-7 and mixed dementia are also shown.

	HR	P	nSNP	L95_HR	U95_HR	Bonferroni-p	Interaction-p
**MODEL 1**							
**PRS 1**	1,08	1,17E-02	16399	1,02	1,15	8,19E-02	
**PRS 2**	1,07	3,93E-02	4896	1,00	1,13	2,75E-01	
**PRS 3**	1,05	1,36E-01	1794	0,99	1,11	9,52E-01	
**PRS 4**	1,04	2,37E-01	882	0,98	1,10	1	
**PRS 5**	1,01	6,72E-01	536	0,95	1,08	1	
**PRS 6**	1,00	9,26E-01	357	0,94	1,06	1	
**PRS 7**	1,00	8,95E-01	253	0,94	1,06	1	
**MODEL 2**							
**PRS 1**	1,17	1,67E-04	16399	1,08	1,26	1,17E-03	1,82E-02
**PRS 2**	1,18	4,75E-05	4896	1,09	1,28	3,33E-04	2,67E-03
**PRS 3**	1,15	6,07E-04	1794	1,06	1,25	4,25E-03	7,98E-03
**PRS 4**	1,13	3,64E-03	882	1,04	1,22	2,55E-02	2,25E-02
**PRS 5**	1,11	9,53E-03	536	1,03	1,20	6,67E-02	1,10E-02
**PRS 6**	1,11	1,24E-02	357	1,02	1,20	8,68E-02	6,28E-03
**PRS 7**	1,10	1,86E-02	253	1,02	1,19	1,30E-01	6,95E-03

***Model 1*:** adjusted for age, sex and education.
***Model 2*:** adjusted for age, sex, education, APOE ε2 and APOE ε4 count (0, 1 or 2 alleles).

Supplementary Table 4. Multivariable Cox regression analyses of associations between PRS 1-7 for type 2 diabetes (standardised) and Alzheimer's disease (AD). Hazard ratios (HR), p-values, number of SNPs (nSNP) and 95% confidence intervals for hazard ratios (L95_HR and U95_HR for lower and upper bounds) are shown for each PRS-score. In Model 2 p-values for interaction between APOE ε4 (0 or 1-2 alleles) and the association between PRS 1-7 and AD are also shown.

	HR	P	nSNP	L95_HR	U95_HR	Interaction_P
**MODEL 1**						
**PRS 1**	1,02	6,26E-01	16396	0,94	1,11	
**PRS 2**	0,98	6,39E-01	4894	0,90	1,06	
**PRS 3**	0,97	5,37E-01	1793	0,90	1,06	
**PRS 4**	0,95	2,17E-01	881	0,88	1,03	
**PRS 5**	0,94	1,44E-01	536	0,87	1,02	
**PRS 6**	0,94	1,38E-01	357	0,87	1,02	
**PRS 7**	0,94	1,55E-01	253	0,87	1,02	
**MODEL 2**						
**PRS 1**	1,05	4,67E-01	16396	0,92	1,19	8,58E-01
**PRS 2**	1,00	9,59E-01	4894	0,88	1,13	6,93E-01
**PRS 3**	0,97	6,81E-01	1793	0,86	1,11	4,25E-01
**PRS 4**	0,98	7,47E-01	881	0,86	1,11	9,34E-01
**PRS 5**	0,98	7,58E-01	536	0,86	1,11	9,86E-01
**PRS 6**	0,99	9,22E-01	357	0,88	1,13	7,91E-01
**PRS 7**	1,02	7,87E-01	253	0,90	1,15	5,33E-01

***Model 1*:** adjusted for age, sex and education.
***Model 2*:** adjusted for age, sex, education, APOE ε2 count, and APOE ε4 count (0, 1 or 2 alleles).

Supplementary Table 5. Multivariable Cox regression analyses of associations between PRS 1-7 for type 2 diabetes (standardised) and vascular dementia (VaD). Hazard ratios (HR), p-values, number of SNPs (nSNP), 95% confidence intervals for hazard ratios (L95_HR and U95_HR for lower and upper bounds), and Bonferroni-corrected p-values are shown for each PRS-score. In Model 2 p-values for interaction between APOE ε4 (0 or 1-2 alleles) and the association between PRS 1-7 and VaD are also shown.

	HR	P	nSNP	L95_HR	U95_HR	Bonferroni-p	Interaction-p
**MODEL 1**							
**PRS 1**	1,13	7,04E-03	16387	1,03	1,23	4,93E-02	
**PRS 2**	1,14	3,62E-03	4888	1,04	1,25	2,53E-02	
**PRS 3**	1,10	3,46E-02	1793	1,01	1,20	2,42E-01	
**PRS 4**	1,09	4,77E-02	882	1,00	1,19	3,34E-01	
**PRS 5**	1,07	1,36E-01	535	0,98	1,17	9,52E-01	
**PRS 6**	1,05	2,77E-01	356	0,96	1,14	1	
**PRS 7**	1,04	3,70E-01	254	0,95	1,13	1	
**MODEL 2**							
**PRS 1**	1,23	2,08E-04	16387	1,10	1,38	1,46E-03	1,89E-02
**PRS 2**	1,28	1,37E-05	4888	1,15	1,43	9,59E-05	2,77E-03
**PRS 3**	1,22	5,41E-04	1793	1,09	1,36	3,79E-03	1,32E-02
**PRS 4**	1,22	4,34E-04	882	1,09	1,36	3,04E-03	5,84E-03
**PRS 5**	1,21	6,26E-04	535	1,09	1,35	4,38E-03	1,56E-03
**PRS 6**	1,19	1,72E-03	356	1,07	1,33	1,20E-02	1,52E-03
**PRS 7**	1,18	2,56E-03	254	1,06	1,32	1,79E-02	1,38E-03

***Model 1*:** adjusted for age, sex and education.
***Model 2*:** adjusted for age, sex, education, APOE ε2 count, and APOE ε4 count (0, 1 or 2 alleles).

Supplementary Table 6. Stratified Analysis for APOE ε4 status (0 or 1-2 alleles): Multivariable Cox regression analyses of associations between PRS 1-7 for type 2 diabetes (standardised) as exposure and all-cause dementia as well as vascular dementia as outcome. Hazard ratio (HR), p-value, and Bonferroni-corrected p-values are shown for each PRS-score. The analysis is adjusted for age, sex and education.

	HR (95% CI)	p	Bonferroni-P
**No ApoE-ε4 (*n* = 20,359)**			
**All-cause dementia**			
**PRS 1**	1.13 (1.06-1.20)	<0.001	7,00E-16
**PRS 2**	1.14 (1.06-1.21)	<0.001	7,00E-16
**PRS 3**	1.09 (1.02-1.16)	0,008	5,60E-02
**PRS 4**	1.06 (1.00-1.13)	0,059	4,13E-01
**PRS 5**	1.05 (0.99-1.12)	0,115	8,05E-01
**PRS 6**	1.06 (1.00-1.13)	0,056	3,92E-01
**PRS 7**	1.07 (1.01-1.14)	0,035	2,45E-01
**VaD**			
**PRS 1**	1.22 (1.09-1.37)	0,001	7,00E-03
**PRS 2**	1.27 (1.13-1.43)	<0.001	7,00E-16
**PRS 3**	1.20 (1.07-1.35)	0,001	7,00E-03
**PRS 4**	1.20 (1.07-1.34)	0,002	1,40E-02
**PRS 5**	1.21 (1.08-1.35)	0,001	7,00E-03
**PRS 6**	1.19 (1.07-1.33)	0,002	1,40E-02
**PRS 7**	1.20 (1.07-1.34)	0,002	1,40E-02
**ApoE-ε4 (*n* = 8,780)**			
**All-cause dementia**			
**PRS 1**	1.01 (0.95-1.07)	0,829	1
**PRS 2**	0.99 (0.93-1.05)	0,737	1
**PRS 3**	1.00 (0.94-1.06)	0,993	1
**PRS 4**	1.00 (0.94-1.06)	0,865	1
**PRS 5**	0.98 (0.92-1.04)	0,47	1
**PRS 6**	0.96 (0.90-1.02)	0,166	1
**PRS 7**	0.96 (0.90-1.02)	0,183	1
**VaD**			
**PRS 1**	1.00 (0.87-1.15)	0,996	1
**PRS 2**	0.98 (0.86-1.13)	0,788	1
**PRS 3**	0.98 (0.86-1.12)	0,767	1
**PRS 4**	0.96 (0.84-1.10)	0,592	1
**PRS 5**	0.91 (0.79-1.04)	0,174	1
**PRS 6**	0.88 (0.77-1.01)	0,079	5,53E-01
**PRS 7**	0.88 (0.76-1.00)	0,057	3,99E-01

Supplementary Table 7. Cox regression analyses of associations between PRS 1-7 of HbA1c (standardised) and all-cause dementia. Model 1: adjusted for age, sex and education. Model 2: adjusted for age, sex, education and APOE- ε4 burden. In Model 2 p-values for interaction between APOE- ε4 and the association between PRS 1-7 and all-cause dementia are also shown.

Model 1	HR	P	nSNP	L95_HR	U95_HR	Interaction_P
**MODEL 1**						
**PRS 1**	0,98	4,53E-01	11675	0,94	1,03	
**PRS 2**	1,00	8,67E-01	2365	0,95	1,04	
**PRS 3**	0,99	7,56E-01	676	0,95	1,04	
**PRS 4**	0,98	3,91E-01	314	0,94	1,02	
**PRS 5**	0,99	5,90E-01	188	0,95	1,03	
**PRS 6**	0,98	3,91E-01	141	0,94	1,02	
**PRS 7**	0,98	4,11E-01	113	0,94	1,03	
**MODEL 2**						
**PRS 1**	1,02	4,41E-01	11675	0,96	1,09	1,59E-01
**PRS 2**	1,05	1,08E-01	2365	0,99	1,12	4,83E-02
**PRS 3**	1,03	4,11E-01	676	0,97	1,09	1,99E-01
**PRS 4**	1,00	9,34E-01	314	0,94	1,06	5,12E-01
**PRS 5**	1,00	9,09E-01	188	0,94	1,07	5,54E-01
**PRS 6**	1,00	8,97E-01	141	0,94	1,06	4,81E-01
**PRS 7**	0,99	8,68E-01	113	0,94	1,06	5,46E-01

Supplementary Table 8. Cox regression analyses of association between PRS 1-7 of HbA1c (standardised) and mixed dementia. Model 1: adjusted for age, sex and education. Model 2: adjusted for age, sex, education and APOE- ε4 burden. In Model 2 p-values for interaction between APOE- ε4 and the association between PRS 1-7 and mixed dementia are also shown.

	HR	P	nSNP	L95_HR	U95_HR	Interaction_P
**MODEL 1**						
**PRS 1**	0,96	2,06E-01	11673	0,91	1,02	
**PRS 2**	0,97	3,53E-01	2367	0,92	1,03	
**PRS 3**	0,96	1,70E-01	676	0,90	1,02	
**PRS 4**	0,95	1,24E-01	315	0,90	1,01	
**PRS 5**	0,97	2,56E-01	189	0,91	1,03	
**PRS 6**	0,96	1,87E-01	141	0,91	1,02	
**PRS 7**	0,96	1,57E-01	113	0,90	1,02	
**MODEL 2**						
**PRS 1**	1,00	9,17E-01	11673	0,92	1,08	3,53E-01
**PRS 2**	1,04	3,13E-01	2367	0,96	1,13	2,42E-02
**PRS 3**	1,00	9,06E-01	676	0,93	1,09	1,10E-01
**PRS 4**	0,99	8,19E-01	315	0,92	1,07	2,00E-01
**PRS 5**	1,00	9,51E-01	189	0,92	1,08	2,78E-01
**PRS 6**	0,99	8,26E-01	141	0,92	1,07	2,44E-01
**PRS 7**	0,99	7,63E-01	113	0,91	1,07	2,54E-01

Supplementary Table 9. Cox regression analyses of associations between PRS 1-7 of HbA1c (standardised) and AD. Model 1: adjusted for age, sex and education. Model 2: adjusted for age, sex, education and APOE- ε4 burden. In Model 2 p-values for interaction between APOE- ε4 and the association between PRS 1-7 and AD are also shown.

Model 1	HR	P	nSNP	L95_HR	U95_HR	Interaction_P
**MODEL 1**						
**PRS 1**	1,01	7,53E-01	11674	0,93	1,10	
**PRS 2**	1,05	2,51E-01	2363	0,97	1,14	
**PRS 3**	1,06	1,83E-01	677	0,97	1,14	
**PRS 4**	1,04	3,50E-01	314	0,96	1,13	
**PRS 5**	1,03	4,66E-01	188	0,95	1,12	
**PRS 6**	1,01	7,37E-01	141	0,94	1,10	
**PRS 7**	1,01	7,42E-01	113	0,94	1,10	
**MODEL 2**						
**PRS 1**	1,07	2,78E-01	11674	0,95	1,22	3,88E-01
**PRS 2**	1,11	1,14E-01	2363	0,98	1,25	3,93E-01
**PRS 3**	1,12	8,67E-02	677	0,98	1,26	2,92E-01
**PRS 4**	1,06	3,52E-01	314	0,94	1,20	6,78E-01
**PRS 5**	1,04	5,71E-01	188	0,91	1,18	9,50E-01
**PRS 6**	1,02	7,54E-01	141	0,90	1,16	8,86E-01
**PRS 7**	1,00	9,60E-01	113	0,88	1,14	8,46E-01

Supplementary Table 10. Cox regression analyses of associations between PRS 1-7 of HbA1c (standardised) and VaD. Model 1: adjusted for age, sex and education. Model 2: adjusted for age, sex, education and APOE- ε4 burden. In Model 2 p-values for interaction between APOE- ε4 and the association between PRS 1-7 and VaD are also shown.

	HR	P	nSNP	L95_HR	U95_HR	Interaction_P
**MODEL 1**						
**PRS 1**	0,95	2,15E-01	11675	0,87	1,03	
**PRS 2**	0,97	4,85E-01	2365	0,89	1,06	
**PRS 3**	0,96	3,81E-01	676	0,88	1,05	
**PRS 4**	0,95	2,85E-01	315	0,88	1,04	
**PRS 5**	0,97	5,17E-01	189	0,89	1,06	
**PRS 6**	0,96	3,73E-01	141	0,88	1,05	
**PRS 7**	0,96	3,08E-01	113	0,88	1,04	
**MODEL 2**						
**PRS 1**	0,99	8,87E-01	11675	0,89	1,11	2,59E-01
**PRS 2**	1,04	4,77E-01	2365	0,93	1,16	5,96E-02
**PRS 3**	1,02	7,61E-01	676	0,91	1,13	1,16E-01
**PRS 4**	1,01	8,57E-01	315	0,91	1,13	1,06E-01
**PRS 5**	1,00	9,78E-01	189	0,90	1,12	4,15E-01
**PRS 6**	0,99	9,20E-01	141	0,89	1,11	3,30E-01
**PRS 7**	0,98	7,31E-01	113	0,88	1,09	4,54E-01

Supplementary Table 11. Cox regression analyses of associations between PRS 1-7 of fasting glucose (standardised) and all-cause dementia. Model 1: adjusted for age, sex and education. Model 2: adjusted for age, sex, education and APOE- ε4 burden. In Model 2 p-values for interaction between APOE- ε4 and the association between PRS 1-7 and all-cause dementia are also shown.

	HR	P	nSNP	L95_HR	U95_HR	Interaction_P
**MODEL 1**						
**PRS 1**	0,99	7,12E-01	7696	0,95	1,04	
**PRS 2**	1,03	2,73E-01	1919	0,98	1,07	
**PRS 3**	0,99	5,02E-01	472	0,94	1,03	
**PRS 4**	0,99	7,79E-01	141	0,95	1,04	
**PRS 5**	1,01	7,70E-01	71	0,96	1,05	
**PRS 6**	1,01	6,66E-01	53	0,97	1,05	
**PRS 7**	1,01	6,66E-01	39	0,97	1,05	
**MODEL 2**						
**PRS 1**	0,97	3,52E-01	7696	0,92	1,03	5,13E-01
**PRS 2**	1,01	8,09E-01	1919	0,95	1,07	6,53E-01
**PRS 3**	0,98	6,05E-01	472	0,93	1,05	7,69E-01
**PRS 4**	1,00	8,90E-01	141	0,94	1,06	8,37E-01
**PRS 5**	1,01	8,48E-01	71	0,95	1,07	9,73E-01
**PRS 6**	1,01	7,16E-01	53	0,95	1,07	9,65E-01
**PRS 7**	1,03	2,81E-01	39	0,97	1,10	2,86E-01

Supplementary Table 12. Cox regression analyses of associations between PRS 1-7 of fasting glucose (standardised) and mixed dementia. Model 1: adjusted for age, sex and education. Model 2: adjusted for age, sex, education and APOE- ε4 burden. In Model 2 p-values for interaction between APOE- ε4 and the association between PRS 1-7 and mixed dementia are also shown.

Model 1	HR	P	nSNP	L95_HR	U95_HR	Interaction_P
**MODEL 1**						
**PRS 1**	0,98	5,40E-01	7693	0,93	1,04	
**PRS 2**	1,05	1,53E-01	1918	0,98	1,11	
**PRS 3**	1,03	2,78E-01	473	0,97	1,10	
**PRS 4**	1,02	5,07E-01	142	0,96	1,08	
**PRS 5**	1,02	5,27E-01	71	0,96	1,08	
**PRS 6**	1,03	3,99E-01	53	0,97	1,09	
**PRS 7**	1,03	3,74E-01	39	0,97	1,09	
**MODEL 2**						
**PRS 1**	0,98	6,82E-01	7693	0,91	1,06	7,36E-01
**PRS 2**	1,05	2,41E-01	1918	0,97	1,14	6,52E-01
**PRS 3**	1,04	3,94E-01	473	0,96	1,12	9,21E-01
**PRS 4**	1,03	4,89E-01	142	0,95	1,11	6,69E-01
**PRS 5**	1,04	3,64E-01	71	0,96	1,12	4,76E-01
**PRS 6**	1,03	4,04E-01	53	0,96	1,12	7,31E-01
**PRS 7**	1,06	1,33E-01	39	0,98	1,15	1,95E-01

Supplementary Table 13. Cox regression analyses of associations between PRS 1-7 of fasting glucose (standardised) and AD. Model 1: adjusted for age, sex and education. Model 2: adjusted for age, sex, education and APOE- ε4 burden. In Model 2 p-values for interaction between APOE- ε4 and the association between PRS 1-7 and AD are also shown.

	HR	P	nSNP	L95_HR	U95_HR	Interaction_P
**MODEL 1**						
**PRS 1**	0,98	6,26E-01	7693	0,91	1,06	
**PRS 2**	1,03	5,05E-01	1920	0,95	1,11	
**PRS 3**	0,98	6,69E-01	473	0,91	1,06	
**PRS 4**	1,00	9,24E-01	142	0,93	1,09	
**PRS 5**	1,01	8,63E-01	71	0,93	1,09	
**PRS 6**	1,00	9,25E-01	53	0,93	1,09	
**PRS 7**	1,01	8,07E-01	39	0,93	1,09	
**MODEL 2**						
**PRS 1**	0,89	7,56E-02	7693	0,79	1,01	7,27E-02
**PRS 2**	0,96	5,02E-01	1920	0,85	1,09	2,00E-01
**PRS 3**	1,02	7,48E-01	473	0,90	1,16	6,22E-01
**PRS 4**	1,02	7,16E-01	142	0,90	1,16	7,18E-01
**PRS 5**	1,00	9,69E-01	71	0,88	1,13	7,30E-01
**PRS 6**	1,02	7,18E-01	53	0,90	1,16	8,16E-01
**PRS 7**	1,06	3,56E-01	39	0,94	1,20	3,89E-01

Supplementary Table 14. Cox regression analyses of associations between PRS 1-7 of fasting glucose (standardised) and VaD. Model 1: adjusted for age, sex and education. Model 2: adjusted for age, sex, education and APOE- ε4 burden. In Model 2 p-values for interaction between APOE- ε4 and the association between PRS 1-7 and VaD are also shown.

	HR	P	nSNP	L95_HR	U95_HR	Interaction_P
**MODEL 1**						
**PRS 1**	0,94	1,80E-01	7696	0,86	1,03	
**PRS 2**	1,05	2,84E-01	1917	0,96	1,15	
**PRS 3**	1,01	7,70E-01	473	0,93	1,11	
**PRS 4**	1,04	4,08E-01	142	0,95	1,13	
**PRS 5**	1,02	5,92E-01	71	0,94	1,12	
**PRS 6**	1,03	4,67E-01	53	0,95	1,13	
**PRS 7**	1,04	4,21E-01	39	0,95	1,13	
**MODEL 2**						
**PRS 1**	0,94	2,44E-01	7696	0,84	1,05	9,73E-01
**PRS 2**	1,08	1,74E-01	1917	0,97	1,21	3,10E-01
**PRS 3**	1,04	5,34E-01	473	0,93	1,16	5,78E-01
**PRS 4**	1,07	2,61E-01	142	0,95	1,19	3,91E-01
**PRS 5**	1,05	4,26E-01	71	0,94	1,17	4,97E-01
**PRS 6**	1,05	4,30E-01	53	0,94	1,17	6,95E-01
**PRS 7**	1,07	2,35E-01	39	0,96	1,19	3,49E-01

Supplementary Table 15. Cox regression analyses of associations between PRS 1-7 of fasting insulin (standardised) and all-cause dementia. Model 1: adjusted for age, sex and education. Model 2: adjusted for age, sex, education and APOE- ε4 burden. In Model 2 p-values for interaction between APOE- ε4 and the association between PRS 1-7 and all-cause dementia are also shown.

	HR	P	nSNP	L95_HR	U95_HR	Interaction_P	Bonferroni_P
**MODEL 1**							
**PRS 1**	0,99	7,10E-01	7675	0,95	1,04		
**PRS 2**	0,99	7,56E-01	1812	0,95	1,04		
**PRS 3**	0,97	2,26E-01	362	0,93	1,02		
**PRS 4**	0,98	3,48E-01	83	0,94	1,02		
**PRS 5**	0,98	2,55E-01	31	0,93	1,02		
**PRS 6**	0,98	4,71E-01	17	0,94	1,03		
**PRS 7**	0,98	3,51E-01	12	0,94	1,02		
**MODEL 2**							
**PRS 1**	1,00	9,59E-01	7675	0,94	1,06	2,05E-01	1
**PRS 2**	0,99	7,56E-01	1812	0,93	1,05	5,09E-01	1
**PRS 3**	0,94	4,25E-02	362	0,88	1,00	9,77E-01	2,98E-01
**PRS 4**	1,00	9,84E-01	83	0,94	1,06	6,11E-01	1
**PRS 5**	0,99	7,13E-01	31	0,93	1,05	8,55E-01	1
**PRS 6**	0,97	3,56E-01	17	0,91	1,03	2,11E-01	1
**PRS 7**	0,96	2,36E-01	12	0,91	1,02	3,78E-01	1

Supplementary Table 16. Cox regression analyses of associations between PRS 1-7 of fasting insulin (standardised) and mixed dementia. Model 1: adjusted for age, sex and education. Model 2: adjusted for age, sex, education and APOE- ε4 burden. In Model 2 p-values for interaction between APOE- ε4 and the association between PRS 1-7 and mixed dementia are also shown.

	HR	P	nSNP	L95_HR	U95_HR	Interaction_P	Bonferroni_P
**MODEL 1**							
**PRS 1**	0,98	5,91E-01	7687	0,93	1,04		
**PRS 2**	0,99	6,31E-01	1812	0,93	1,05		
**PRS 3**	0,94	4,47E-02	362	0,89	1,00		
**PRS 4**	0,98	5,10E-01	83	0,92	1,04		
**PRS 5**	0,97	3,80E-01	31	0,92	1,03		
**PRS 6**	0,97	3,03E-01	17	0,91	1,03		
**PRS 7**	0,98	5,05E-01	12	0,92	1,04		
**MODEL 2**							
**PRS 1**	0,95	1,79E-01	7687	0,88	1,02	4,17E-01	1
**PRS 2**	0,93	7,18E-02	1812	0,86	1,01	9,35E-02	5,03E-01
**PRS 3**	0,88	1,65E-03	362	0,81	0,95	1,90E-01	1,16E-02
**PRS 4**	0,95	2,21E-01	83	0,88	1,03	1,81E-01	1
**PRS 5**	0,97	4,51E-01	31	0,90	1,05	5,10E-01	1
**PRS 6**	0,94	1,01E-01	17	0,86	1,01	7,42E-02	7,07E-01
**PRS 7**	0,97	3,92E-01	12	0,89	1,05	5,37E-01	1

Supplementary Table 17. Cox regression analyses of associations between PRS 1-7 of fasting insulin (standardised) and AD. Model 1: adjusted for age, sex and education. Model 2: adjusted for age, sex, education and APOE- ε4 burden. In Model 2 p-values for interaction between APOE- ε4 and the association between PRS 1-7 and AD are also shown.

	HR	P	nSNP	L95_HR	U95_HR	Bonferroni_P	Interaction_P
**MODEL 1**							
**PRS 1**	0,99	7,30E-01	7676	0,91	1,07	1	
**PRS 2**	1,02	5,76E-01	1812	0,95	1,11	1	
**PRS 3**	1,02	6,91E-01	362	0,94	1,10	1	
**PRS 4**	0,92	5,58E-02	83	0,85	1,00	3,91E-01	
**PRS 5**	0,92	3,39E-02	31	0,85	0,99	2,37E-01	
**PRS 6**	0,95	1,89E-01	17	0,87	1,03	1	
**PRS 7**	0,94	1,31E-01	12	0,87	1,02	9,17E-01	
**MODEL 2**							
**PRS 1**	1,05	4,50E-01	7676	0,93	1,19	1	7,21E-02
**PRS 2**	1,09	1,78E-01	1812	0,96	1,22	1	7,11E-02
**PRS 3**	1,05	4,85E-01	362	0,92	1,19	1	8,33E-02
**PRS 4**	0,99	9,08E-01	83	0,87	1,13	1	2,99E-01
**PRS 5**	0,95	3,72E-01	31	0,83	1,07	1	9,36E-01
**PRS 6**	0,94	3,76E-01	17	0,83	1,07	1	5,62E-01
**PRS 7**	0,86	1,57E-02	12	0,76	0,97	1,10E-01	4,32E-02

Supplementary Table 18. Cox regression analyses of associations between PRS 1-7 of fasting insulin (standardised) and VaD. Model 1: adjusted for age, sex and education. Model 2: adjusted for age, sex, education and APOE- ε4 burden. In Model 2 p-values for interaction between APOE- ε4 and the association between PRS 1-7 and VaD are also shown.

	HR	P	nSNP	L95_HR	U95_HR	Interaction_P
**MODEL 1**						
**PRS 1**	1,01	8,74E-01	7685	0,92	1,10	
**PRS 2**	1,00	9,89E-01	1811	0,92	1,09	
**PRS 3**	0,95	2,40E-01	362	0,87	1,04	
**PRS 4**	0,96	3,95E-01	83	0,88	1,05	
**PRS 5**	0,94	1,96E-01	31	0,87	1,03	
**PRS 6**	0,93	1,05E-01	17	0,85	1,02	
**PRS 7**	0,96	3,25E-01	12	0,88	1,04	
**MODEL 2**						
**PRS 1**	1,00	9,73E-01	7685	0,90	1,12	8,89E-01
**PRS 2**	1,01	8,51E-01	1811	0,91	1,13	5,61E-01
**PRS 3**	0,92	1,65E-01	362	0,83	1,03	8,87E-01
**PRS 4**	0,96	4,89E-01	83	0,86	1,07	8,72E-01
**PRS 5**	0,96	4,33E-01	31	0,86	1,07	8,80E-01
**PRS 6**	0,93	1,87E-01	17	0,83	1,04	7,17E-01
**PRS 7**	0,96	4,30E-01	12	0,86	1,07	9,22E-01

Supplementary Table 19. Comparison of PRS models with variants at Minor allele frequency of 0.01 and 0.05 for the Multivariable Cox regression analyses of associations between PRS 1-7 of type 2 diabetes (standardised) and all-cause dementia. Hazard ratios with corresponding p-values and number of SNPs (nSNP) are shown. In Model 2 p for interaction between APOE- ε4 and the association between type 2 diabetes and all-cause dementia is also shown.

	HR	P	nSNPs	Interaction-p
**MAF 0.01, Model 1**				
**PRS 1**	1,05	2,70E-02	30743	
**PRS 2**	1,04	5,89E-02	7607	
**PRS 3**	1,03	2,00E-01	2404	
**PRS 4**	1,01	8,17E-01	1074	
**PRS 5**	0,99	7,68E-01	603	
**PRS 6**	0,98	4,51E-01	400	
**PRS 7**	0,99	5,84E-01	282	
**MAF 0.05, Model 1**				
**PRS 1**	1,05	2,87E-02	16405	
**PRS 2**	1,03	1,60E-01	4891	
**PRS 3**	1,02	4,51E-01	1795	
**PRS 4**	1,00	9,54E-01	882	
**PRS 5**	0,99	5,28E-01	536	
**PRS 6**	0,98	3,38E-01	357	
**PRS 7**	0,98	4,02E-01	253	
**MAF 0.01, Model 2**				
**PRS 1**	1,11	7,78E-04	30743	4,54E-02
**PRS 2**	1,11	1,09E-03	7607	6,07E-02
**PRS 3**	1,09	8,54E-03	2404	9,76E-02
**PRS 4**	1,06	4,42E-02	1074	5,25E-02
**PRS 5**	1,06	7,04E-02	603	5,73E-02
**PRS 6**	1,05	8,40E-02	400	3,26E-02
**PRS 7**	1,06	5,26E-02	282	2,99E-02
**MAF = 0.05, Model 2**				
**PRS 1**	1,11	5,57E-04	16405	4,36E-02
**PRS 2**	1,11	5,12E-04	4891	2,11E-02
**PRS 3**	1,08	1,19E-02	1795	1,07E-01
**PRS 4**	1,07	3,43E-02	882	6,43E-02
**PRS 5**	1,06	7,48E-02	536	5,80E-02
**PRS 6**	1,06	5,01E-02	357	1,57E-02
**PRS 7**	1,07	2,80E-02	253	1,10E-02

***Model 1*:** adjusted for age, sex and education.
***Model 2*:** adjusted for age, sex, education, APOE ε2 count, and APOE ε4 count (0, 1 or 2 alleles).

Supplementary Table 20. Mendelian randomisation (MR) with type 2 diabetes as exposure (243 SNPs as instrumental variables) and dementia types as outcome variables, using different mathematical models of causal effect estimates (MR Egger, weighted median, inverse variance weighted, simple mode and weighted mode). Beta (causal effect size), standard error and p-value are shown for each MR-method and outcome.

Outcome	Method	Beta	SE	p
**All-cause dementia**	MR Egger	-0,08	0,2	6,77E-01
**All-cause dementia**	Weighted median	0,003	0,1	9,75E-01
**All-cause dementia**	Inverse variance weighted	-0,04	0,1	6,14E-01
**All-cause dementia**	Simple mode	0,07	0,2	6,90E-01
**All-cause dementia**	Weighted mode	0,04	0,1	7,12E-01
**Mixed dementia**	MR Egger	0,02	0,2	9,17E-01
**Mixed dementia**	Weighted median	0,09	0,1	3,71E-01
**Mixed dementia**	Inverse variance weighted	0,01	0,1	9,40E-01
**Mixed dementia**	Simple mode	0,09	0,3	7,05E-01
**Mixed dementia**	Weighted mode	0,09	0,2	5,19E-01
**AD**	MR Egger	-0,15	0,2	5,36E-01
**AD**	Weighted median	-0,13	0,1	1,24E-01
**AD**	Inverse variance weighted	-0,11	0,1	2,59E-01
**AD**	Simple mode	-0,11	0,2	6,24E-01
**AD**	Weighted mode	-0,11	0,1	3,89E-01
**VaD**	MR Egger	0,06	0,2	7,80E-01
**VaD**	Weighted median	0,15	0,1	2,89E-01
**VaD**	Inverse variance weighted	0,1	0,1	2,75E-01
**VaD**	Simple mode	-0,11	0,4	7,61E-01
**VaD**	Weighted mode	0,1	0,2	6,10E-01

Supplementary Table 21. Mendelian randomization for the APOE ε4 negative group, with type 2 diabetes as exposure (243 SNPs as instrumental variables) and dementia sub-types as outcomes, using different mathematical models of causal effect estimates (MR Egger, weighted median, inverse variance weighted, simple mode and weighted mode). Beta (causal effect size), standard error and p-value are shown for each MR-method and outcome.

Outcome	Method	Beta	SE	p
**All Cause Dementia**	Inverse variance weighted	-0,13	0,08	4,29E-01
**All Cause Dementia**	MR Egger	0,06	0,19	4,51E-01
**All Cause Dementia**	Simple mode	0,14	0,19	4,58E-01
**All Cause Dementia**	Weighted median	0,12	0,08	2,21E-01
**All Cause Dementia**	Weighted mode	-0,27	0,11	6,03E-02
**Mixed Dementia**	Inverse variance weighted	0,005	0,08	6,17E-01
**Mixed Dementia**	MR Egger	0,03	0,19	5,96E-01
**Mixed Dementia**	Simple mode	0,14	0,25	3,37E-01
**Mixed Dementia**	Weighted median	-0,14	0,10	6,11E-01
**Mixed Dementia**	Weighted mode	-0,23	0,15	1,26E-01
**AD**	Inverse variance weighted	0,11	0,10	4,37E-01
**AD**	MR Egger	0,02	0,24	6,11E-01
**AD**	Simple mode	0,19	0,22	2,32E-01
**AD**	Weighted median	-0,31	0,09	2,61E-01
**AD**	Weighted mode	-0,23	0,13	1,89E-01
**VaD**	Inverse variance weighted	-0,06	0,09	3,89E-01
**VaD**	MR Egger	0,14	0,22	2,41E-01
**VaD**	Simple mode	0,07	0,35	6,27E-01
**VaD**	Weighted median	0,12	0,14	4,90E-01
**VaD**	Weighted mode	-0,23	0,20	3,04E-01
